# Peripheral endothelial function and arterial stiffness in patients with chronic migraine: a case–control study

**DOI:** 10.1186/1129-2377-14-8

**Published:** 2013-01-31

**Authors:** Pedro Enrique Jiménez Caballero, Francisco Muñoz Escudero

**Affiliations:** 1 Department of Neurology, San Pedro de Alcántara Hospital, Avenida Pablo Naranjo 2. 1003, Cáceres, Spain; 2 Department of Neurology, Virgen de la Salud Hospital, Avenida de Barber 32, Toledo, 45005, Spain

**Keywords:** Chronic migraine, Endothelial dysfunction, Case control study, EndoPAT device arterial stiffness

## Abstract

**Background:**

Migraine is a complex and disabilitating neurovascular disorder predominantly affecting women. There is strong evidence that nitric oxide is critically involved in migraine pathophysiology. The aim of the present study was to test the hypothesis that chronic migraine is associated with ultrasonographic endothelial dysfunction and increase in arterial stiffness. These parameters were assessed using a novel plethysmograph by peripheral arterial tonometry.

**Methods:**

Twenty-one patients with chronic migraine and twenty-one healthy controls matched by sex and gender were recruited. Measurement of the ultrasonographic endothelial function and augmentation index were made according to manufacturer’s protocol.

**Results:**

The mean of patient’s peripheral arterial tonometry ratios was 1.93 ± 0.39 and for healthy control 2.21 ± 0.44 (p = 0.040). The median of patients’ augmentation index was −6,0 (IQR: 6.5 to −15) in healthy controls and 9.0 (IQR: 4 to 12) in chronic migraine, (p = 0.002).

**Conclusions:**

Patients with chronic migraine have ultrasonographic endothelial dysfunction and increase in the arterial stiffness. An improved understanding of the role in the endothelial system of migraine may provide a basis for preventive drugs in migraine and restore the endothelial function.

## Background

Migraine is a complex and debilitating neurovascular disorder predominantly affecting women. It recurs as attacks of severe headache associated with nausea, vomiting, phonophobia, and photophobia. In some cases, migraine headache is preceded by transient neurologic symptoms that are known as aura. According to the second edition of the International Classification of Headache Disorders (ICHD-II) [1] chronic migraine (CM) is defined as at least 15 days of headache per month over the past months, of which at least eight headache days meet the criteria for migraine without aura or respond to migraine-specific treatment. The prevalence rate of CM is estimated to be 1.4% to 2.2% of the general population [2].

Despite its high prevalence, migraine is a disease involving complex pathogenetic mechanisms that remain to be clarified [3]. However, there is strong evidence that nitric oxide (NO) is critically involved in migraine pathophysiology [4]. NO plays an essential role in the control of cerebral blood flow and may be involved in the activation of nociceptors in the trigeminovascular system and release of vasoactive neuropeptides during neurogenic inflammatory response [5]. Endothelial dysfunction is commonly described as the inability of the artery to sufficiently dilate in response to an appropriate endothelial stimulus, but also comprises endothelial activation, which is characterized by proinflammatory and procoagulatory milieu. By ultrasound it can be assessed by measurement of the arterial pulse wave at a finger artery or by the measurement of flow-mediated dilation (FMD) of the brachial artery after occlusion of the blood flow. FMD has several disadvantages: it is operator dependent and as FMD is measured at one arm only, there are not possibilities to correct for potential measurement-induced changes in the systemic hemodynamics, such as those resulting from alterations in the autonomous nervous system tone. To overcome these problems, the EndoPAT was developed. This device detects plethysmographic pressure changes in the finger tips caused by the arterial pulse and translates this to a peripheral arterial tone (PAT). Endothelium-mediated changes in vascular tone after occlusion of the brachial artery are reflecting a downstream hyperemic response, which is measure for arterial endothelial function [6]. Measurements on the contralateral arm are used to control for concurrent nonendothelium-dependent changes in vascular tone. In addition, the EndoPAT provides a measure for arterial stiffness: the augmentation index (AI). Several EndoPAT studies have demonstrated an improvement in ultrasonographic endothelial function as a result of lifestyle modification [7] or prolonged pharmacological intervention [8].

The aim of the present study was to test the hypothesis that chronic migraine is associated with ultrasonographic endothelial dysfunction and an increase in arterial stiffness using a novel non-invasive peripheral finger plethysmograph (EndoPAT). We study the chronic migraines because is probably than the changes in the ultrasonographic endothelial dysfunction were more important and persistent than in episodic migraines.

## Methods

### Study population

Patients with CM were consecutively recruited from the outpatient headache clinic between May 2009 and September 2010. Twenty-one patients with CM who fulfilled the diagnostic criteria of CM according to the ICHD-II were included. All patients had detailed clinical evaluation and a structured interview about history of headache by an experienced neurologist. Twenty-one healthy participants were recruited from volunteers among the staff of the hospital and served as control group. Healthy volunteers without history of migraine were matched with patients by sex and age. Exclusion criteria were age < 18 and ≥ 50 years, body mass index (BMI) < 18 and ≥ 30 kg/m2, history of cardiovascular disease, Raynaud syndrome, peripheral vascular disease, inflammatory conditions, active cancer, ovarium pathology, smoking, arterial hypertension (systolic blood pressure > 140 mmHg or diastolic blood pressure > 90 mmHg), diabetes mellitus (fasting plasma glucose ≥ 126 mg/dl), hypercholesterolemia (> 220 mg/dl), pregnancy or lactation and regular use of vasoactive drugs. Sporadic consumptions of vasoactive drugs for more than a week before the test were allowed. We reject to make the EndoPAT test in subject with morphologic defects or finger wounds. Carotid intima-media thickness (IMT) was measured in patients and controls. The first author made the interviews and the test, so he was not blinded to the patient or control subjects. The hospital Ethics Committee approved the study protocol and all patients gave informed consent.

### Study protocol

**Figure 1:**
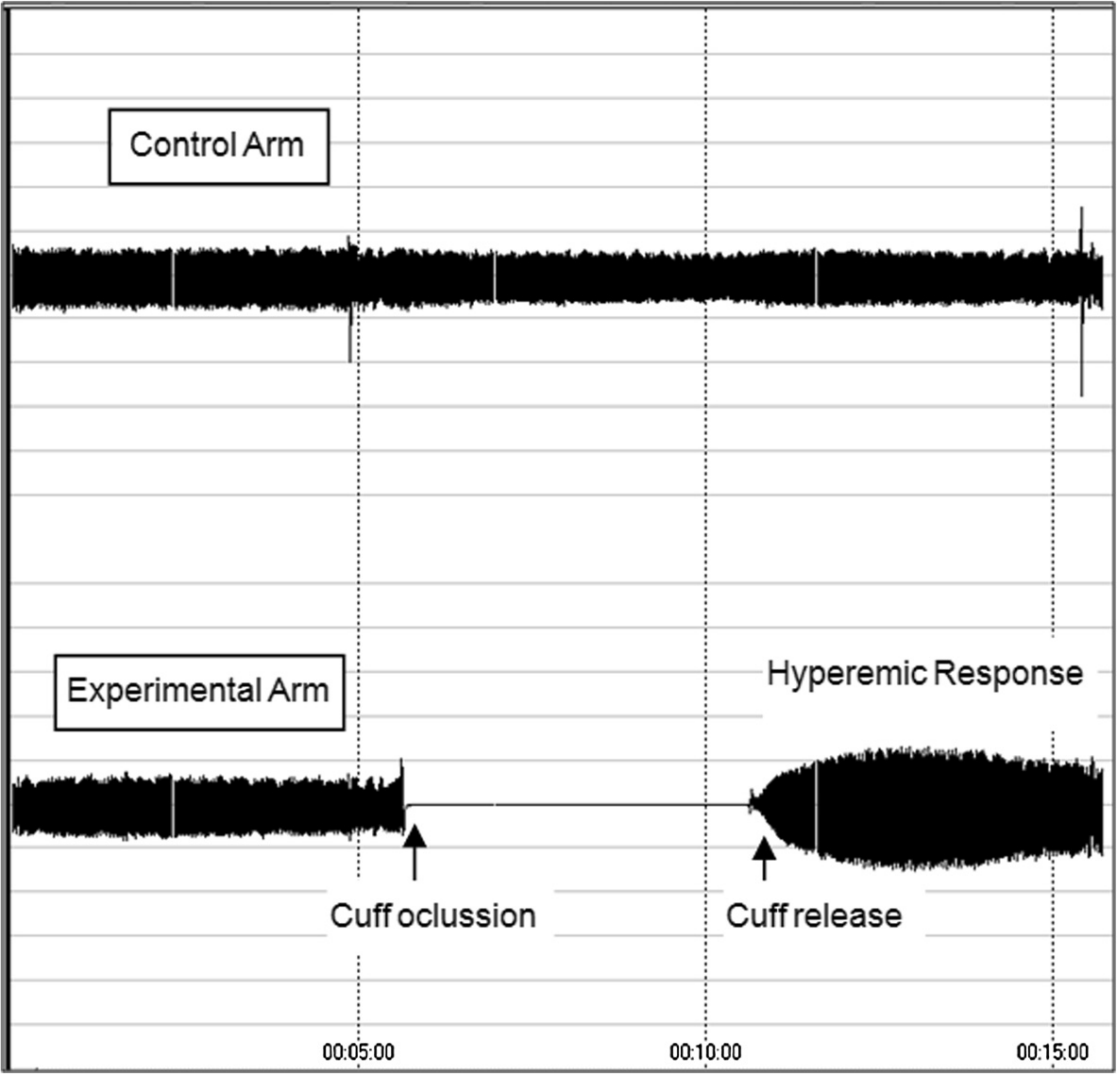
Representative peripheral arterial tonometry recordings of reactive hyperaemic response after 5 minutes of occlusion of the braquial artery.

Reactive hyperaemia index (RHI), which is a measure of endothelial function, and augmentation index (AI), which is a measure for arterial stiffness, were assessed using the EndoPAT 2000 device (Itamar, Israel). AI is the difference between the first and the second peaks of the arterial waveform. Values are expressed as a percentage of the central pulse pressure. In addition, because AI is influenced by changes in heart rate, all values of AI were corrected to 75 beats per minute (AI@75 bpm) [9]. Assessment of the ultrasonographic endothelial function was based on the measurement of peripheral arterial tonometry (PAT) of the index finger during reactive hyperaemic of the forearm vascular bed as previously described [6]. All measures were calculated using a computerised automated algorithm provided with the device. Measurements were performed according to manufacturer’s instructions [10]. Briefly, the subjects were in supine position for a minimum of 20 minutes before measurements, in a quiet, temperature-controlled (21-24°C) room with dimmed lights. The subjects were asked to remain as still as possible and silent during the entire measurement period. Each recording consisted of 5 minutes of baseline measurement, 5 minutes of occlusion measurement, and 5 minutes postocclusion measurement (hyperaemic period) as is showed in the Figure 1. Occlusion of the brachial artery was performed on the nondominant upper arm. The occlusion pressure was at least 60 mmHg above the systolic blood pressure (minimally 200 mmHg, and maximally 300 mmHg).

IMT was defined according to the Mannheim Consensus as the distance between the characteristic echoes of the lumen-intima interface and the media-adventitia interface for both sides.

### Statistical analysis

Sample size estimation was based on a previous study that investigated differences in endothelial-dependent FMD between migraine and healthy controls [11], an alpha error of 0.05 and a power of the test of the 80%. We check the sample for a normal distribution of observed RHI and AI values. Data are expressed as mean ± SD or median and interquartile range (IQR) for continuous variables and as frequencies and proportions for categorical variables. Continuous data from the two groups were compared using properly the Mann–Whitney *U*-test or unpaired *t*-test. For correlation the Spearman rank correlation was used. All tests were two-tailed, and statistical significance was determined at an alpha level of 0.05. Statistical analyses were performed using SPSS version 15.0.

## Results

**Figure 2:**
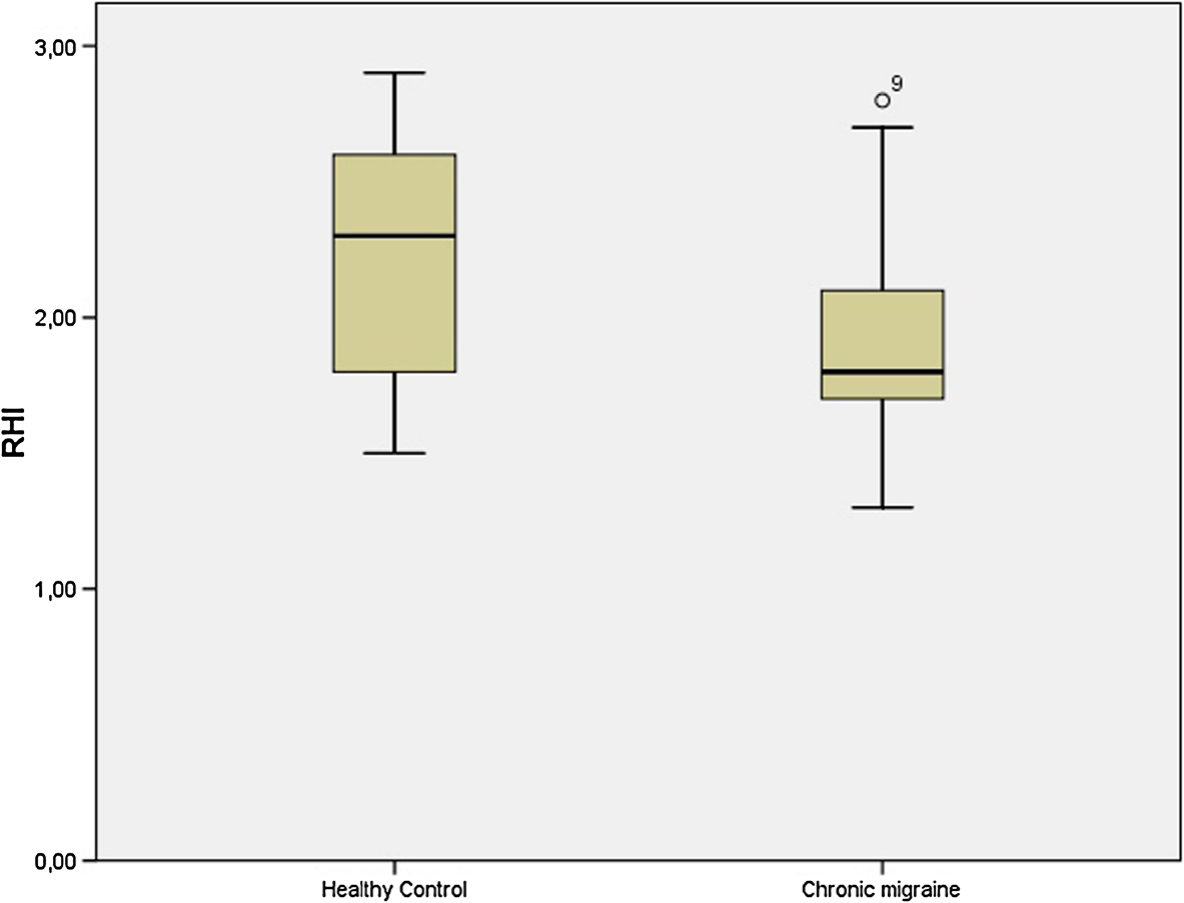
Blox-plot that shows the differences in the RHI (expressed as ratios) between chronic migraine patients and healthy controls.

**Figure 3:**
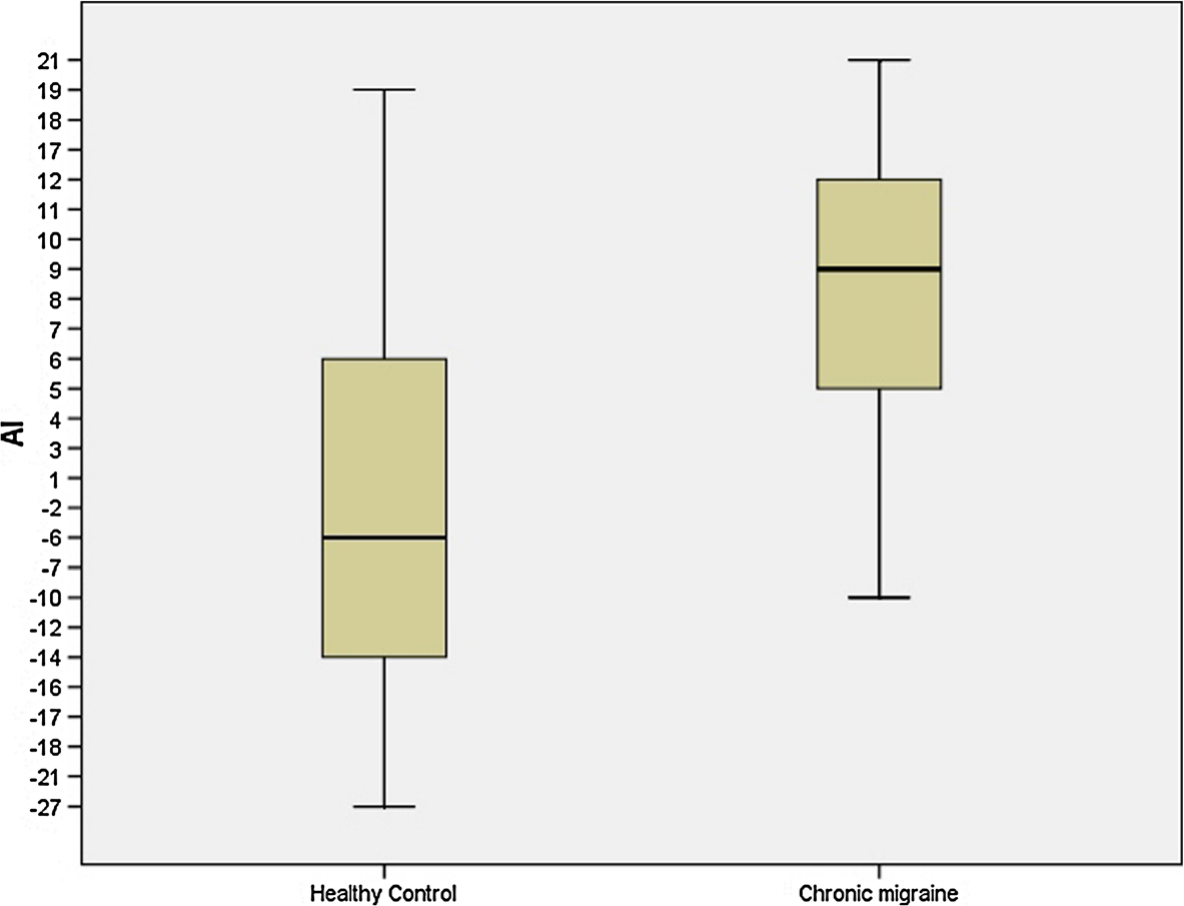
Box-plot that shows the differences in the AI (expressed as percentages) between chronic migraine patients and healthy controls.

Twenty-one patients with CM were enrolled in this study. 6 of them were males (28.5%) and 15 were females (71.4%). The number of days per month with headache was 21.4 ± 5.2. The mean of patients’ age was 36.05 ± 7.32 (with a range of 18 to 49 years) and for healthy control was 36.29 ± 6.83 (p: 0.914). The mean of patients’ BMI was 24.52 ± 2.31 and for healthy control was 23.76 ± 2.54 (p = 0.514). The mean of patients’ IMT was 0.68 ± 0.008 and for healthy control was 0.69 ± 0.007 (p = 0.712). The mean of patients’ PAT ratios was 1.93 ± 0.39 and for healthy control 2.21 ± 0.44 (p = 0.040) (Figure 2). The median of patients’ heart-rate-average AI [median and IQR] was of −6.0 (IQR: 8.5 to −17) in healthy controls and 9.0 (IQR: 4 to 12) in chronic migraine, (p = 0.002) (Figure 3). The median of patients’ heart-rate-adjusted AI@75 was −3.0 (IQR: 10 to −18) in healthy controls and 8.0 (IQR: 5.5 to 14.5) in chronic migraine, (p = 0.004).

There were not differences in the RHI by gender: females 2.08 ± 0.47 and males 2.05 ± 0.35 (p = 0.870). There was not correlation between RHI and age (R = −0.132, p = 0.405) or BMI (R = +0.126, p = 0.412).

There was not differences in the AI by gender: females −4 (IQR: 5 to −16) and males −7 (IQR: 12 to −21) (p = 0.253). There were not correlation between AI and age (R = −0.055, p = 0.727), number of days with headache (R = +0.073; p = 0.628) or BMI (R = +0.187, p = 0.235). There were not correlation between RHI and number of days per month with headache (R:-0,218; p = 0.083). There was a negative correlation between RHI and heart-rate-average AI (R = −0.377, p = 0.014).

## Discussion

Our study shows the following findings: 1) AI as an indirect marker for arterial stiffness is increased in patients with CM compared with matched healthy controls; and 2) patients with CM have worse ultrasonographic endothelial function using digital reactive hyperaemic peripheral arterial tonometry.

Evidence of endothelial dysfunction in migraine is increasing. Migraine is associated with reduction in the number and function of endothelial progenitor cells, serving as a marker for dysfunction of the endothelium [12] and its counts decrease as migraine progresses in time [13]. In this same study didn’t found differences in the FMD between patients with and healthy control. They suggest a relation with the intensity of headaches more than its frequency.

Some migraine patients could to have an arterial super-sensitivity to NO [14] may be explained by an autonomic dysfunction. Some authors [15] reported a possible impairment of sympathetic control of cerebral blood flow in migraine patients.

Previous studies have already investigated the ultrasonographic endothelial function and NO vascular response describing a normal [16-18], reduced [19,20] or increased FMD [21] in migraine patients respect to controls. However, two studies evaluated only patients affected by migraine without aura [18,19], and two studies did not distinguish between migraine with aura or without aura in their analytic approach [16,20].

A recent study using EndoPAT device did not find evidence for systemic endothelial dysfunction in migraine with aura patients [22]. The mean of attacks per month was 3.2. In the same study, the authors found an augmentation in the arterial stiffness measured by AI. Probably, the difference in the number of attacks per month respect to our study could determinate the lack of alteration in the ultrasonographic endothelial function.

Endothelial function as measured by the EndoPAT could be physiologically different from endothelial function as measured by the conventional techniques [23]. Onkelinx et al. [24] studied the reproducibility of different methods to measure the endothelial function and found that the within-day variability was lower for the FMD measurements than for the PAT measurements, but the between-day variability was similar.

Some studies [25,26] have shown that patients with migraine have higher IMT than healthy controls and suggest that migraine could be a risk factor for atherosclerosis, but our study don’t found differences. Malik et al. [27] have shown in healthy controls a negative correlation between vascular reactivity and arterial stiffness. Our too have shown this relation in patients with CM.

There are some reports indicating association with migraine and other vascular related situations. Prevalence of migraine has been found to be higher in patients with vasospastic angina than in controls [28]. Miller et al. [29] reported that the prevalence of migraine was 26% in 62 patients with variant angina, which was higher than that in a coronary control group (6%). Smyth et al. [30] reported than patients with primary Raynoud’s phenomenon, there is a higher personal history of migraine than in controls. Nagai et al. [31] have found that migraine is associated with enhanced arterial stiffness.

Our study has several limitations. Firstly, AI is not a direct measure of arterial stiffness. It has been suggested that arterial stiffness and augmented pressure from wave reflexions significantly contribute to the digital volume pulse inflection point; however, AI is influenced by several other factors, such as gender differences in vascular geometry, peripheral resistance, height, and pulse wave velocity. Secondly, for practical reasons, the investigator who performed the measurements was not blinded to the subjects’ history. However, all on-line measurements were completely automated and off-line calculations and analyses were performed blinded after encoding the original files. Thirdly, given the small sample size in our study, our findings and its generalizability have to be interpreted with caution, and further evidence from larger observational studies is needed. Finally, we did not report data from the presence or not of headache during the realization of the probe, sporadic consumptions of vasoactive drugs for more than a week before the test and neither from the duration of the disease. These data could be important to understand the endothelial function in migraine.

One study revelled that Enalapril can possibly improve endothelial function [32]. An improved understanding of the role in the endothelial system of migraine may provide a basis for preventive drugs in migraine [33] and restore the endothelial function.

## Conclusions

Patients with chronic migraine habe ultrasonographic endothelial dysfunction and increase in the arterial stiffness. An improved understanding of the role in the endothelial system of migraine may provide a basis for preventive drugs in migraine and restore the endothelial function.

## Competing interests

There are not conflicts of interests among the authors.

## Authors’ contributions

PEJC was in charge of the recluitment of patients with chronic migraine and the carrying out the studies of pletismography. FME was in charge of putting the patients in the database and assist in the preparation of the manuscript. All the authors read and approved the final manuscript.
